# Children’s Internet Use, Self-Reported Life Satisfaction, and Parental Mediation in Europe: An Analysis of the EU Kids Online Dataset

**DOI:** 10.3389/fpsyg.2021.698176

**Published:** 2022-01-11

**Authors:** Tijana Milosevic, Seffetullah Kuldas, Aikaterini Sargioti, Derek A. Laffan, James O’Higgins Norman

**Affiliations:** ^1^DCU Anti-Bullying Centre, Institute of Education, Dublin City University, Dublin, Ireland; ^2^Department of Media and Communication, University of Oslo, Oslo, Norway

**Keywords:** life satisfaction, Internet use, parental mediation, children, wellbeing

## Abstract

The present research examines how children’s time spent online is associated with their perceived life satisfaction accounting for their age, gender, socio-economic status (SES), emotional problems, country, and family environmental factors. This article is based on the data of the large scale cross-sectional *EU Kids Online* survey from 16 European countries with nationally representative samples of children aged 9–17 (*N* = 11,200, *M*_*age*_ = 13.3, SD = 2.36; 50.6% boys, 49.4% girls). The results indicated that the time children spent online appeared to have no considerable negative effect on their self-reported life satisfaction (SRLS). Comparatively, the positive effects of children’s SES and family environment accounted for 43% of the overall 50% of the variance in children’s SRLS scores. Considering that children’s SES alone accounted for 42% of the variance, children’s emotional problems, country of residence, and enabling parental mediation accounted for the remaining 3, 4, and 1% of the variance, respectively. In line with previous studies that urge caution when discussing the negative influence of time spent online on children’s mental health and overall wellbeing, the current findings suggest that social-ecological characteristics and how children use the Internet, need to be examined further.

## Introduction

Internet technology and digital media have become central to children’s home, school, and social lives. This is perhaps best evidenced in recent times during the COVID-19 crisis (Drouin et al., 2020). It is therefore becoming more important to understand how these technologies are affecting the mental health and life satisfaction of children and young people. The ongoing debate about the mental health impact of Internet and smartphone use on children and youth is considerably polarised. On the one hand, some studies have suggested that the use of the Internet, smartphone, and social media, constitutes an important factor in the rising levels of anxiety, depression, and suicidal ideation for those born after the year 2000, the so-called iGeneration or Post Millennials (Twenge, 2014; Twenge et al., 2020, 2018a,b). On the other hand, more recent longitudinal studies have demonstrated contrasting evidence that substantiates claims for the negative impact often lack robustness (Przybylski and Weinstein, 2017; Heffer et al., 2019; Orben and Przybylski, 2019; Orben et al., 2019).

There have also been concerns that the Internet, mobile devices, and social media are detracting from children’s sleeping patterns, physical activity, and limit abilities to socially engage in a meaningful and empathetic way due to overreliance on technology (Turkle, 2017; Bozkurt et al., 2018; Kobak et al., 2018; Xu et al., 2019). Simultaneously, “social media addiction,” “social media disorder”, as well as the “Internet addiction disorder” have become established terms in the literature as well as the media (Van Den Eijnden et al., 2016; Blackwell et al., 2017; Hawi et al., 2019), bringing Internet use closer to the pathological discourses in mental illness diagnostic criteria such as the Diagnostic and Statistical Manual of Mental Disorders (DSM-5).^1^ In DSM 5, for instance, Internet Gaming Disorder is listed as a condition in need of further clinical research, and the possibility of its inclusion has raised concerns in the scientific community around stigmatising game play and Internet use generally; and not being able to establish the distinction between excessive but non-problematic game play, from the one that contributes to impairments in one’s daily life (Kuss et al., 2017; Parekh, 2018). Other concerns about Internet use and children’s wellbeing include online safety and a variety of risks such as cyberbullying, grooming, sexting, and exposure to harmful content, among others (Smahel et al., 2020). Children’s use of smartphone technology has been especially debated in the context of negative effects, with research findings that an increasing reliance on smartphones contributes to declining mental health among children and adolescents as well as behavioural problems (Twenge et al., 2018b).

Commonplace in several popular media outlets, debates about the problematic term of “screen time” (Hiniker et al., 2019) and children’s Internet use in general, can subject digital and Internet technologies to moral and media panic situations (Drotner, 1999; Buckingham and Jensen, 2012; Staksrud and Ólafsson, 2020). Such discourse promotes emerging technologies as inherently and morally “bad” for children, and implicitly assumes a direct causal link between the use and the negative implications. These debates often discuss children in developmental terms, failing to acknowledge that children can have agency and potentially become architects of positive own digital lives (Staksrud and Livingstone, 2009).

In the subsequent sections, we further evaluate the conceptions of self-assessed subjective wellbeing and life satisfaction, and parental mediation, in order facilitate the justification for the empirical study that follows.

### Self-Reported Wellbeing and Life-Satisfaction: Definitions and Correlates

Subjective (self-reported) wellbeing (SWB) has cognitive, affective, (Diener, 2000; Ben-Zur, 2003; Bradshaw et al., 2011), and social dimensions (Zhang et al., 2019). The affective dimension refers to positive (pleasant) and negative (unpleasant) affects, whereas the cognitive dimension is the “judgement of individual’s life qualities” (Bradshaw et al., 2011, pp. 548–549). Hence, SWB is indicated by life satisfaction, positive affect, negative affect (Diener et al., 1985), and meaning in life in a social-ecological context (Zhang et al., 2019). This characterises SWB as a long-term evaluation of one’s own life (Andrews and Withey, 1976; Keyes et al., 2002; Bradshaw et al., 2011). Therefore, SWB can be measured either in a specific domain like a social network, family, or school (Zhang et al., 2019) or as a global assessment of life satisfaction (Diener et al., 1985). The *EU Kids Online* survey measures the cognitive component only as a self-reported global assessment of life satisfaction (in relation to no specific domain). Given that self-reported mental health also strongly contributes to self-reported life satisfaction (SRLS) (Lombardo et al., 2018), the current analysis further focused on children’s “emotional problems” as another correlate of the SRLS.

Among social factors, large differences in SWB have been reported at the national level, suggesting that one’s life circumstances and country-level factors such as socio-economic and ethno-cultural differences, play a significant role (Inglehart and Klingemann, 2000; White, 2010). Likewise, such country characteristics can also make a substantial difference in the extent to which children can capitalise on various online opportunities, and how they experience online risks and possible harms (Staksrud and Milosevic, 2017). Some studies found that country-associated characteristics are likely to account for children’s SWB substantially, while other socio-demographic characteristics like child’s age and gender, are likely to have a much smaller contribution (Dinisman and Ben-Arieh, 2016). However, these socio-demographic variables are very likely to be correlated with children’s time spent online. For instance, recent research on children’s Internet use and wellbeing has found that girls are disproportionately negatively affected, suggesting the need for further research on gender differences (Twenge et al., 2020).

A recent study, which conducted cross- and within-country comparisons, as to whether the amount of time children spend online affects their overall life satisfaction in developing countries, found no strong association and also no evidence that this association differs by gender (Kardefelt-Winther et al., 2020). In the cited study, the quality of children’s close relationships with their family and friends had much stronger explanatory power for their life satisfaction than did time spent on the Internet. Therefore, focusing on the quality of children’s relationships with their family and friends, rather than on restrictions of time children spent online, is likely to explain more of their overall SWB (Kardefelt-Winther et al., 2020). Recent studies have thereby called for more international research to examine the determinant role of family and social context in children’s SWB (Matin et al., 2017; Kardefelt-Winther et al., 2020). It is also important to highlight the problematic nature of the term “screen time” and that not all screen time is created equal; i.e., it is not just the number of hours spent online that matters; but which activities the child engages in online and how this time is spent (Kucirkova and Livingstone, 2017).

### Family Environment as Restrictive and Enabling Parental Mediation

Following the call for additional research into the role of family context in understanding the effect of time spent online on children’s wellbeing, we take into account whether and how restrictive and enabling parental mediation of children’s Internet use affect children’s SRLS. There is a limited amount of research on the relationship between parental mediation of children’s digital media use and their wellbeing (Zullig et al., 2005; Brooks et al., 2016); and mediation specifically has, to our best knowledge, not been studied as a determinant of SRLS, and not when demographic, psychological and country level factors have been accounted for, which is the contribution of our research. Parental or caregiver mediation can affect children’s experience of being online, the incidence of risks and consequent harm, as well as the development of digital skills, which all contribute to children’s subjective wellbeing (Livingstone and Helsper, 2008; Paus-Hasebrink et al., 2013; Livingstone et al., 2017; Livingstone et al., 2018). Parental or caregiver mediation of children’s Internet use refers to parents’ verbal and technical strategies aimed at maximising online opportunities and minimising online risks (Livingstone et al., 2017). Parental mediation can be either parent or child-initiated communication about children’s media and technology use, including activities, content, and time spent online (Sasson and Mesch, 2014, 2019; Kuldas et al., 2021). Building on the earlier work on parental mediation of child Internet use (Sasson and Mesch, 2014, 2019; Livingstone et al., 2017), and in an effort to reconcile inconsistencies in scales used to measure parental mediation, researchers have most recently proposed a trichotomy of restrictive-enabling-observant parental mediation of children’s Internet use (Kuldas et al., 2021). According to this model, restrictive mediation refers to parental controls in forms of (a) setting rules for their children’s use of social media, apps, or games, (b) setting filters (technical restriction to online contents), and (c) setting limits (the Internet access), while also monitoring/checking their children’s online activities (Kuldas et al., 2021). Enabling mediation includes parent- and child-initiated communications (sharing, asking, talking, listening, discussing, or encouraging) and instructions (helping, explaining, showing, suggesting, or guiding) that enable children’s agency in Internet and social media use, managing online safety/risks and opportunities (Kuldas et al., 2021). Observant mediation is when a parent intermittently observes or attentive to both the child’s behaviour and the screen when the child is online (Kuldas et al., 2021).

Previous research has found that different mediation strategies are associated with differences in family characteristics when it comes to children’s exposure to online risks and opportunities (Livingstone et al., 2017). For instance, restrictive mediation was preferred when child and parent digital skills were lower; it largely contributed to the reduction of online risks but also diminished the exposure to online opportunities, such as learning or improving digital skills (Livingstone et al., 2017). Enabling mediation, on the other hand, was preferred by parents who were more digitally savvy; it increased more online opportunities but also risks (Livingstone et al., 2017). Parental mediation strategies also appeared to be associated with family climate: parents who exhibited higher confidence in their parenting skills were more likely to rely on active mediation in the form of co-use with their child, which in turn positively influenced the family climate (Festl and Gniewosz, 2019). Having in mind the role of restrictive mediation in reducing risk exposure, and the effect of enabling mediation on maximising opportunities and the possible influence over family climate, we found it important to account for the role of parental mediation of Internet use in determining children’s SRLS.

### The Current Study

We seek to understand if, and to what extent, time spent online plays a role in determining SRLS when demographic, psychological, family and country-level factors have been accounted for. We investigate the following research questions:

1. Does time children spend online explain their overall SRLS levels when children’s gender, age, socio-economic status (SES), emotional problems, and country of residence have been accounted for?
2. How does children’s family environment (i.e., enabling and restrictive parental mediation of their Internet use) affect children’s SRLS when the above factors have been accounted for?

## Materials and Methods

The *EU Kids Online*^2^ study is a multinational research network that sought to enhance knowledge of children’s online opportunities, risks and safety across 19 European countries over the course of 2018 and 2019. This research network consists of the undertaking of large scale surveys and qualitative inquiry to act as a basis of evidence for the purposes of informing safer Internet policies that promote online safety for children in Europe. In the present study, the secondary data analysis, as of the *EU Kids Online* survey data, includes nationally representative samples of Internet using children aged 9–17 of 16 European countries (*N* = 11,200). The recommended sample size as directed by the network was 1,000 children per country with a few exceptions for smaller populated countries. See Table 1 for sample description and the descriptive statistics across measured variables.

**TABLE 1:** Descriptive statistics of demographic, independent, and dependent variables.

Variables	*n*	%	*M*	SD
Gender				
Boys	5670	50.6		
Girls	5530	49.4		
Country				
Czechia	1290	11.5		
Estonia	577	5.2		
Finland	357	3.2		
France	658	5.9		
Germany	751	6.7		
Italy	621	5.5		
Lithuania	450	4.0		
Malta	553	4.9		
Norway	785	7.0		
Poland	531	4.7		
Portugal	1086	9.7		
Romania	447	4.0		
Serbia	660	5.9		
Slovakia	575	5.1		
Spain	1524	13.6		
Switzerland	335	3.0		
Age			13.3	2.36
Socio-economic Status			6.76	1.62
Emotional Problems			1.84	0.72
Enabling Mediation			2.7	1.0
Restrictive Mediation			3.29	0.89
Internet Use			5.4	1.64
Life Satisfaction			7.45	1.63

*N = 11,200.*

The *EU Kids Online* survey contained core items that all countries needed to include, such as: questions about child’s Internet and digital technology use patterns, risks (e.g., cyberbullying, sexting, and grooming), psychological characteristics, family environment/parental mediation, and well-being. There were also optional questions about children’s Internet and digital media use. Accordingly, the length of the survey varied by country.

Each country followed ethics procedures directed by the *EU Kids Online* network and sought approval or advice from its relevant national institution or ethical committee, if available. Informed consent from both parents/caregivers and children was sought ensuring confidentiality and anonymity. For a more detailed description of the sampling methods, data and the research process including the ethics procedures please consult the *EU Kids Online* technical report (Zlamal et al., 2020).

### Dependent Variable

The *EU Kids Online* study measured overall life satisfaction (*M* = 7.45, SD = 1.63) with the Cantril Ladder that reads as follows: “Here is a picture of a ladder. Imagine that the top of the ladder ‘10’ is the best possible life for you and the bottom ‘0’ is the worst possible life for you. In general, where on the ladder do you feel you stand at the moment? Please tick the box next to the number that best describes where you stand.” At the top of the ladder, it was written: “Best possible life” and the bottom of the ladder there was the label “Worst possible life.”

### Independent Variables

#### Internet Use

The *EU Kids Online* survey measured children’s estimated Internet use (i.e., time spent online and intensity). Two questions assessed the amount of estimated time children spent online on weekdays and on weekends on a nine-point-scale ranging from 1 (little or no time), 2 (about half an hour), 3 (one hour), 4 (two hours), 5 (three hours), 6 (four hours), 7 (five hours), 8 (six hours), to 9 (seven hours or more). The question that measures estimated time spent on the Internet is intended to capture the difference in use between weekdays when children’s daily schedule is constricted by school activities, and weekends when they tend to have more free time. To measure *intensity of Internet use*, the *EU Kids Online* survey asked children to rate how often they go online or use the internet via four devices (i.e., a mobile phone/smartphone, desktop computer, laptop/notebook, and tablet) on a seven-point-scale ranging from 0 (never) to 7 (almost all the time).

A Principal Component Analysis (PCA) of a correlation matrix of these six items measuring children’s Internet use (either alone or along with nine items measuring family environment, restrictive and enabling mediation) suggested to retain only three items: two items measuring time spent online, and one item measuring the intensity of “a mobile phone/smartphone” use (see Table 2). The other three items (i.e., desktop computer, laptop/notebook, and tablet) were not factorable and had very low loading values well below 0.30. Therefore, these three items were excluded from further analysis. The Internet use/time spent online had high internal consistency with a composite reliability value of 0.85 (*M* = 5.4, SD = 1.64) well above the cut-off values of 0.70 (Fornell and Larcker, 1981).

**TABLE 2:** Results from the principal Component Analysis of the EUKO questionnaire.

Item	Component loading		
	1	2	3
**Factor 1: Family Environment—Enabling Mediation and Child Initiated Mediation**			
Helps me when something bothers me on the internet	0.806		
Suggests ways to use the internet safely	0.789		
Talks to me about what I do on the internet	0.761		
Told my parent or carer about things that bother or upset me on the internet	0.750		
Asked for my parent’s or carer’s help with a situation on the internet that I could not handle	0.714		
Encourages me to explore and learn things on the internet	0.676		
**Factor 2: Family Environment—Restrictive Mediation**			
Download music or films		0.809	
Use a web or phone camera		0.796	
Use a social networking site		0.791	
**Factor 3: Internet Use—Time Spent Online and Intensity of Use**			
During a regular weekend-day			0.886
During a regular weekday (school-day)			0.876
A mobile phone or smartphone			0.648
Eigenvalues	3.95	2.5	1.20
% of variance	32.9	20.6	10
Composite reliability	0.89	0.84	0.85

*N = 11,200. The extraction method was Principal Component Analysis with orthogonal (varimax with Kaiser normalisation) rotation. Component loadings <0.32 were omitted.*

### Psychological Characteristics

Psychological characteristics were initially assessed through a set of 10 core items, adapted from the Strengths and Difficulties Questionnaire (Dew and Huebner, 1994; Goodman et al., 1998) that measure conduct problems (three items), emotional problems (four items), and hyperactivity (three items) on a 4-point-scale: not true (1), a bit true (2), fairly true (3), very true (4) for me. Negatively worded items were reversed and analysed accordingly. However, only the emotional problems scale had good internal consistency with a composite reliability value of 0.78 (*M* = 1.84, SD = 0.72). Due to their very low internal consistency with composite reliability value of 0.49 for conduct problems scale and 0.05 for the hyperactivity scale (i.e., below the cut-off values of 0.70, Fornell and Larcker, 1981), they were excluded from further analysis.

### Socio-Demographic Variables

The analysis included three demographic variables: age (*M* = 13.3, SD = 2.36), gender (boys set as the reference category), and SES. Children reported their SES based on the question: “Think of this ladder as representing where people stand in your country. At the top of the ladder are the people who are the best off—those who have the most money, the most education and the most respected jobs. At the bottom are the people who are the worst off—who have the least money, the least education, and the least respected jobs or no job. Please tick the box where you think you and your family are” (Zlamal et al., 2020).

### Family Environment as Parental Mediation Strategies

Indicators of family environment were restrictive and enabling parental mediation strategies for child’s Internet use (see Table 2 for the results of PCA). Enabling mediation (converged with Child-Initiated Mediation), was measured via six core items with a 5-point scale from “1 = never” to “5 = very often.” Restrictive mediation was measured with three core items on a 4-point scale (1 = I am allowed to do this anytime, 2 = I am allowed to do this only with permission or supervision, 3 = I am not allowed to do this, and 4 = I do not know if I am allowed to do this). Both enabling and restrictive mediation scales had high internal consistency with a composite reliability value of 0.89 (*M* = 2.7, SD = 1.0) and 0.84 (*M* = 3.29, SD = 0.89) above the cut-off values of 0.70 (Fornell and Larcker, 1981), respectively.

### Country-Level Variable

Table 1 shows the list of countries included in the study. In a hierarchical multiple regression, a variable with the largest sample size is automatically considered as a reference to other variables, which in this case was Spain.

### Data Analyses and Procedures

The current study carried out two main statistical analyses using IBM SPSS v.27 statistical software. First, a PCA of family environment (restrictive and enabling mediation) and child’s Internet use identified an optimal number of components indicating parental mediation of child’s Internet use. The PCA was conducted on the 14 items with an orthogonal (varimax) rotation method, because the primary purpose was to retain significantly correlated items which converge into fewer components. Results of this PCA allowed for further analysis of the second research question. Second, a Hierarchical Multiple Regression analysis of children’s SRLS was conducted to account for explanatory effects of the Internet use, while controlling for children’s gender, age, SES, emotional problems, and country. These demographic variables were first introduced, followed by emotional problems, which are the strongest predictors of SWB (Diener et al., 1999; Bradshaw et al., 2011; Kim et al., 2019), then country level (Dinisman and Ben-Arieh, 2016), time spent online, and finally parental mediation variables.

Prior to conducting the regression analysis, plots of standardised residuals to evaluate for assumptions of normality, homoscedasticity, linearity, multicollinearity, independence of errors, and absence of outliers were checked (Tabachnick and Fidell, 2014). All the statistical assumptions were met for the analysis.

## Results

### A Principal Component Analysis of Items Measuring Parental Mediation of Child’s Internet Use

Based on the results of the PCA, a total of two items with a loading less than 0.32 was removed, as they were below the cut-off value (Tabachnick and Fidell, 2014). For the remaining 12 items, the Kayser–Meyer–Olkin measure verified the sampling adequacy for the analysis (KMO = 0.83). The Bartlet test was significant (*p* < 0.001). An initial analysis was run to obtain eigenvalues for each component in the data. Three components had eigenvalues over Kaiser’s criterion of 1 and in combination explained 63.5% of the variance. The first component accounted for 32.9%, the second accounted for 20.6%, and the third component accounted for 10% of the variance. Table 2 displays the items and the rotated component loadings, of which less than 0.32 were omitted to improve clarity. The items that cluster on the same component suggest that Component 1 represented Family Environment—Enabling Mediation with six items (including Child Initiated Mediation with two items), Component 2 represented Family Environment—Restrictive Mediation with three items, and Component 3 represented the Internet Use (consisted of Time Spent Online with two items and Intensity of Use with one item). All the components had high values of internal consistency, estimated with composite reliability of 0.89, 0.84, and 0.85, respectively (Table 2).

### A Hierarchical Multiple Regression Analysis of Children’ Self-Reported Life Satisfaction

A 5-step hierarchical multiple regression was conducted to estimate the prediction of children’s perceived life satisfaction based on their gender, age, and SES (Step 1); emotional problems, one indicator of psychological wellbeing (Step 2); country, namely Czechia, Estonia, Finland, France, Germany, Italy, Lithuania, Malta, Norway, Poland, Portugal, Romania, Serbia, Slovakia, Spain, and Switzerland (Step 3); internet use (Step 4); and family environment, enabling mediation and restrictive mediation (Step 5). As shown in Table 3, Step 1 included gender, age, and SES as controlling for variables in the model, which accounted for 42% of the variation in perceived life satisfaction, *R*^2^ = 0.42, *F*(3, 112) = 2745.43, *p* < 0.001. Step 2 added emotional problems (a variable of psychological wellbeing) to the model, which increased 3% and accounted for 45% in the variation in perceived life satisfaction, *R*^2^ = 0.45, *F*(4, 112) = 2300.95, *p* < 0.001. Step 3, the inclusion of 16 countries (i.e., Czech, Estonia, Finland, France, Germany, Italy, Lithuania, Malta, Norway, Poland, Portugal, Romania, Serbia, Slovakia, Spain, and Switzerland) in the model increased 4% and accounted for 49% in the variation in perceived life satisfaction, *R*^2^ = 0.49, *F*(19, 112) = 556.40, *p* < 0.001. Step 4, the addition of internet use to the model made no considerable contribution to the accounted 49% of the variance in perceived life satisfaction, *R*^2^ = 0.49, *F*(20, 112) = 530.59, *p* < 0.001. The last Step 5 included two enabling mediation and restrictive mediation as the family environment factors, which increased only 1% variation in the model. As a result, all together the 22 independent variables accounted for 50% in the variation in children’s’ perceived life satisfaction, *R*^2^ = 0.50, *F*(22, 112) = 508.43, *p* < 0.001.

**TABLE 3:** Hierarchical multiple regression results for predictors of children’s perceived life satisfaction.

Variables	B	95% CI for B		SE B	β	*R* ^2^	Δ*R*^2^
		LL	UP				
Step 1						0.42	0.42***
Constant	4.78***	4.60	4.95	0.09			
Gender	−0.15***	–0.19	–0.10	0.02	–0.05		
Age	−0.11***	–0.12	–0.10	0.01	–0.16		
Socio-economic Status	0.62***	0.60	0.63	0.01	0.61		
Step 2						0.45	0.03***
Constant	5.35***	5.17	5.53	0.09			
Gender	–0.02	–0.07	0.03	0.02	–0.01		
Age	−0.09***	–0.10	–0.08	0.01	–0.13		
Socio-economic Status	0.60***	0.59	0.62	0.01	0.60		
Emotional Problems	−0.39***	–0.42	–0.36	0.02	–0.17		
Step 3						0.49	0.04***
Constant	5.62***	5.44	5.81	0.09			
Gender	–0.01	–0.05	0.04	0.02	–0.00		
Age	−0.08***	–0.09	–0.07	0.01	–0.12		
Socio-economic Status	0.61***	0.60	0.62	0.01	0.60		
Emotional Problems	−0.46***	–0.50	–0.43	0.02	–0.21		
Czechia	−0.13**	–0.22	–0.04	0.05	–0.03		
Estonia	−0.48***	–0.59	–0.36	0.06	–0.06		
Finland	−0.31***	–0.45	–0.18	0.07	–0.03		
France	−0.84***	–0.94	–0.73	0.06	–0.12		
Germany	−0.81***	–0.91	–0.70	0.05	–0.12		
Italy	−0.33***	–0.44	–0.22	0.07	–0.05		
Lithuania	−0.80***	–0.93	–0.67	0.06	–0.10		
Malta	−0.22***	–0.33	–0.10	0.06	–0.03		
Norway	−0.41***	–0.51	–0.31	0.05	–0.06		
Poland	−0.66***	–0.77	–0.54	0.06	–0.09		
Portugal	–0.05	–0.14	0.05	0.05	–0.01		
Romania	0.11	–0.01	0.24	0.06	0.01		
Serbia	−0.16**	–0.26	–0.05	0.06	–0.02		
Slovakia	−0.83***	–0.94	–0.72	0.06	–0.11		
Switzerland	−0.27***	–0.41	–0.13	0.07	–0.03		
Step 4						0.49	0.00***
Constant	5.66***	5.48	5.84	0.09			
Gender	–0.01	–0.05	0.04	0.02	–0.00		
Age	−0.07***	–0.08	–0.06	0.01	–0.10		
Socio-economic Status	0.61***	0.60	0.62	0.01	0.60		
Emotional Problems	−0.45***	–0.49	–0.42	0.02	–0.20		
Czechia	−0.13**	–0.22	–0.04	0.05	–0.03		
Estonia	−0.48***	–0.59	–0.36	0.06	–0.06		
Finland	−0.30***	–0.44	–0.16	0.07	–0.03		
France	−0.86***	–0.97	–0.75	0.06	–0.12		
Germany	−0.82***	–0.93	–0.72	0.05	–0.13		
Italy	−0.35***	–0.46	–0.24	0.06	–0.05		
Lithuania	−0.81***	–0.93	–0.68	0.06	–0.10		
Malta	−0.21***	–0.33	–0.10	0.06	–0.03		
Norway	−0.40***	–0.50	–0.29	0.05	–0.06		
Poland	−0.67***	–0.78	–0.55	0.06	–0.09		
Portugal	–0.05	–0.14	0.04	0.05	–0.01		
Romania	0.11	–0.02	0.23	0.06	0.01		
Serbia	−0.15*	–0.25	–0.04	0.06	–0.02		
Slovakia	−0.85***	–0.97	–0.74	0.06	–0.12		
Switzerland	−0.28***	–0.42	–0.14	0.07	–0.03		
Internet Use	−0.04***	–0.05	–0.02	0.01	–0.04		
Step 5						0.50	0.01***
Constant	4.87***	4.67	5.08	0.11			
Gender	−0.05*	–0.10	–0.01	0.02	–0.02		
Age	−0.06***	–0.07	–0.05	0.01	–0.09		
Socio-economic Status	0.60***	0.58	0.61	0.01	0.59		
Emotional Problems	−0.46***	–0.49	–0.43	0.02	–0.20		
Czechia	−0.11*	–0.20	–0.03	0.04	–0.02		
Estonia	−0.50***	–0.61	–0.39	0.06	–0.07		
Finland	−0.32***	–0.46	–0.19	0.07	–0.04		
France	−0.98***	–1.09	–0.87	0.06	–0.14		
Germany	−0.83***	–0.93	–0.72	0.05	–0.13		
Italy	−0.41***	–0.52	–0.30	0.06	–0.06		
Lithuania	−0.87***	–0.99	–0.74	0.06	–0.10		
Malta	−0.30***	–0.41	–0.19	0.06	–0.04		
Norway	−0.30***	–0.43	–0.18	0.06	–0.05		
Poland	−0.66***	–0.78	–0.55	0.06	–0.09		
Portugal	−0.13**	–0.22	–0.03	0.05	–0.02		
Romania	0.05	–0.07	0.17	0.06	0.01		
Serbia	−0.23***	–0.33	–0.12	0.06	–0.03		
Slovakia	−0.90***	–1.01	–0.79	0.06	–0.12		
Switzerland	−0.27***	–0.41	–0.13	0.07	–0.03		
Internet Use	−0.03***	–0.05	–0.02	0.01	–0.03		
Enabling Mediation	0.21***	0.18	0.23	0.01	0.12		
Restrictive Mediation	0.06***	0.02	0.09	0.02	0.03		

N = 11,200; CI, confidence interval; LL, lower limit; UL, upper limit. *p < 0.05. **p < 0.01. ***p < 0.001.

## Discussion

The aim of this study was to examine the extent to which time spent online explains children’s SRLS when demographic, psychological, family and country-level factors have been accounted for. We carried out a hierarchical multiple regression model using factorial variables obtained from the *EU Kids Online* large-scale dataset of Internet using children (*N* = 11,200) in 16 European countries.

Following previous literature, the analysis included demographic characteristics (i.e., gender, age, SES, and country of residence) as well as self-reported emotional problems of children, all of which were found to be related to children’s SRLS (Diener et al., 1999; Bradshaw et al., 2011; Dinisman and Ben-Arieh, 2016; Lombardo et al., 2018; Kim et al., 2019). The analysis also examined how enabling and restrictive parental mediation of children’s Internet use (Helsper et al., 2013; Paus-Hasebrink et al., 2013; Livingstone et al., 2017; Kardefelt-Winther et al., 2020) affected children’s SRLS. Providing further inferential statistical evidence for the association between children’s Internet use and their self-reported wellbeing, this analysis of the *EU Kids Online* data, may facilitate consistency in findings which have appeared contradictory in the growing body of research. While some studies found Internet and digital media contribute to negative psychological outcomes such as depression, anxiety, lower levels of empathy, and physical decline (Twenge, 2014; Turkle, 2017; Bozkurt et al., 2018; Kobak et al., 2018; Twenge et al., 2018a,b; Xu et al., 2019), this set of findings found limited support in other studies (Przybylski and Weinstein, 2017; Heffer et al., 2019; Orben et al., 2019; Orben and Przybylski, 2019; Kardefelt-Winther et al., 2020).

The current findings indicate that the estimated time children spent online has no considerable negative effect on their SRLS. When added to the overall model, Internet use made no observable changes in the variation. Children’s self-reported SES and family environment together explained 43% of the positive variance, while their self-reported emotional problems and country of residence accounted for the remaining three per cent and four per cent of variance, respectively. This finding is, to some extent, contrary to previous research which found that demographic variables had a limited contribution to the overall variance while a greater portion was explained by country-level variables (Dinisman and Ben-Arieh, 2016).

While children’s age and gender variables became significant in the final model, SES consistently contributed to SRLS. For higher SES, the probability of a higher level of children’s SRLS increased. However, higher levels of emotional problems decreased the probability of children’s perceived life satisfaction. Likewise, the greater the Internet use (i.e., for every additional hour spent online on smartphone during regular weekdays or weekends), the probability of children’s perceived life satisfaction decreased. Nonetheless, adding Internet use to the model did not increase the explained variance in SRLS. In contrast, both enabling and restrictive parental mediation increased the probability of children’s SRLS.

This study underscores the importance of understanding the relationship between time children spent online and the family context, especially parental/caregiver mediation. Previous research has indicated that restrictive parental mediation strategies can reduce children’s exposure to risks, but also to positive opportunities for children to capitalise on while using the Internet, such as learning, digital skills, socialisation, and leisure (Livingstone et al., 2017). According to previous literature, restrictive mediation, which involves active engagement in child’s Internet use, tended to be employed when both parents and children exhibited less developed digital skills, whereas enabling mediation was employed by parents who had higher levels of digital prowess (Livingstone et al., 2017). Importantly, parents who felt more confident in their skills also tended to rely more on active mediation and co-use behaviour, which was found to positively influence family climate (Festl and Gniewosz, 2019).

While the current finding for the positive effect of enabling and restrictive parental mediation of child Internet use on children’s SWB allows for no further conclusions, other parent-child characteristics (e.g., parent–child engagement in physical activity after or before an online activity) are worth exploring in more detail. More active family involvement in enabling mediation, which facilitates children’s ability to capitalise on online opportunities, might have a more significant bearing on children’s SRLS. This is in contrast to a simple reduction of risk experiences facilitated by restrictive mediation.

## Conclusion

The present research documented empirical evidence for the two main questions. Evidence for the first question indicated that the time children spent online had no considerable negative effect on their SRLS, while taking into account their demographic variables. As evidence for the second question, SES, emotional problems, and country of residence accounted for their SRLS more than family environment.

The cross-sectional nature of this study limits the possibility of a causal or linear relationship of children’s Internet use with their self-reported SWB. Moreover, as SWB has been operationalised across studies in different ways, this study operationalised children’s SRLS as one dimension of their SWB. While our research can contribute to the wider body of work on children’s SWB, this contribution relates only to measures of SRLS and not the affective and domain-specific components outlined in the literature review.

Furthermore, this study measures time spent online only and it does not examine children’s activities (e.g., social media use, online gaming, or learning) nor does it examine online risks (e.g., cyberbullying, sexting etc.). While the inclusion of such variables was beyond the scope of this article, previous research has called for an examination of specific children’s online experiences, rather than merely time spent online. Future research might examine specific online activities in connection to family context and mediation variables. Although parental mediation strategies were examined in the current research, they were identified on the basis of a PCA, which is an item reduction method that can lead to an overestimation of factor loadings (Cabrera-Nguyen, 2010). Instead of PCA, a method of common factor analysis like Principal Axis Factoring or Maximum Likelihood can serve as a precursor of confirmatory factor analysis (CFA) of parental mediation strategies (Kuldas et al., 2021).

The findings in this study arguably provide a strong case for future researchers to pursue additional research into the role of contextual factors and family environment specifically in children’s SRLS. Mediator and moderator effects of enabling and restrictive parental mediation should be explored; especially accounting for other variables such parent–child relationships and parental support; online risks, activities, and digital skills. As the presence of children becomes more prominent with the online world, so are more efforts being made by researchers, parents, policy makers, educators, and technologists who strive to maximise the benefits that the online world brings to children, all the while minimising the potential risks attributed to it.

## Data Availability Statement

Data were accessed through the EU Kids Online network, information available here: https://www.lse.ac.uk/media-and-communications/research/research-projects/eu-kids-online/toolkit.

## Ethics Statement

For information on ethics, please consult the EU Kids Online network’s technical report which details all the procedures concerning ethical aspects of data collection in all participating countries, available here: http://eprints.lse.ac.uk/105835/. Written informed consent was provided by one of participants’ parents/guardians and participating children provided informed assent.

## Author Contributions

TM led the project conception, conceptualization, theoretical direction, interpretation of the statistical analyses and results, and original draft preparation. SK was involved in the conceptualization, theoretical direction, and original draft preparation, carried out the main inferential statistical analyses, and contributed to the overall discussion of the findings. AS carried out the main inferential statistical analyses and contributed to the overall discussion of the findings. DL wrote up the preliminary descriptive results and contributed to the discussion of findings. JO’H contributed to the supervision and the writing—reviewing and editing of this project. All authors contributed to the article and approved the submitted version.

## Conflict of Interest

The authors declare that the research was conducted in the absence of any commercial or financial relationships that could be construed as a potential conflict of interest.

## Publisher’s Note

All claims expressed in this article are solely those of the authors and do not necessarily represent those of their affiliated organizations, or those of the publisher, the editors and the reviewers. Any product that may be evaluated in this article, or claim that may be made by its manufacturer, is not guaranteed or endorsed by the publisher.

## Footnotes

1
https://www.psychiatry.org/psychiatrists/practice/dsm/feedback-and-questions/frequently-asked-questions#::~text=The%20Diagnostic%20and%20Statistical%20Manual,criteria%20for%20diagnosing%20mental%20disorders

2
https://www.lse.ac.uk/media-and-communications/research/research-projects/eu-kids-online
